# Intracellular immunization against HIV infection with an intracellular antibody that mimics HIV integrase binding to the cellular LEDGF protein

**DOI:** 10.1038/s41598-017-16742-2

**Published:** 2017-12-04

**Authors:** Leyuan Bao, Clare Hannon, Abimael Cruz-Mignoni, Denis Ptchelkine, Mei-yi Sun, Ami Miller, Wilawan Bunjobpol, Camilo E. Quevedo, Mariliza Derveni, Jennifer Chambers, Alison Simmons, Simon E. V. Phillips, Terence H. Rabbitts

**Affiliations:** 1 Weatherall Institute of Molecular Medicine, MRC Molecular Haematology Unit, University of Oxford, John Radcliffe Hospital, Oxford, OX3 9DS UK; 2 School of Clinical Medicine, University of Cambridge, Addenbrooke’s Hospital, Hills Rd, Cambridge, CB2 0SP UK; 3 Research Complex at Harwell, Rutherford Appleton Laboratory, Didcot, OX11 0FA UK; 4 Weatherall Institute of Molecular Medicine, MRC Human Immunology Unit, University of Oxford, John Radcliffe Hospital, Oxford, OX3 9DS UK; 5 Department of Biochemistry, University of Oxford, South Parks Road, Oxford, OX1 3QU UK

## Abstract

Preventing the protein-protein interaction of the cellular chromatin binding protein Lens Epithelium-Derived Growth Factor (LEDGF) and human immunodeficiency virus (HIV) integrase is an important possible strategy for anti-viral treatment for AIDS. We have used Intracellular Antibody Capture technology to isolate a single VH antibody domain that binds to LEDGF. The crystal structure of the LEDGF-VH complex reveals that the single domain antibody mimics the effect of binding of HIV integrase to LEDGF which is crucial for HIV propagation. CD4-expressing T cell lines were constructed to constitutively express the LEDGF-binding VH and these cells showed interference with HIV viral replication, assayed by virus capsid protein p24 production. Therefore, pre-conditioning cells to express antibody fragments confers effective intracellular immunization for preventing chronic viral replication and can be a way to prevent HIV spread in infected patients. This raises the prospect that intracellular immunization strategies that focus on cellular components of viral integrase protein interactions can be used to combat the problems associated with latent HIV virus re-emergence in patients. New genome editing development, such as using CRISPR/cas9, offer the prospect intracellularly immunized T cells in HIV+ patients.

## Introduction

Protein-protein interactions (PPIs) are important inside cells for transcription, signaling and formation of complex organelles and a range of normal cellular activities such as maintenance, control of cell division, and quiescence. If these become abnormal in disease states, the modulation of the PPIs can be important for therapy. An important mediator of MLL (mixed lineage leukaemia; HGNC nomenclature KMT2A) protein function and of HIV productive infection is LEDGF (Lens Epithelium-Derived Growth Factor), (HGNC nomenclature PSIP1, also known as PC4 and SFRS1 interacting protein) functions by binding MLL but also Human Immunodeficiency Virus type 1 (HIV-1) productive infection through interaction with HIV-1 Integrase (IN). The main functions of LEDGF are in transport and tethering other proteins to chromatin. The N-terminal portion of the protein carries a nuclear localization sequence that binds to chromatin, and another domain called the Integrase Binding Domain (IBD) that binds to MLL and Menin (MEN1) reviewed in^1^.

Advances in anti-retroviral therapy (ART) have transformed HIV/AIDS from a deadly pandemic to a treatable but long-term chronic disease^2^. ART has contributed to reduction in HIV replication at the individual level and in subsequent transmission at a population level. This has raised optimism that Treatment-as-Prevention (TasP) may arrest the global pandemic by 2020. A barrier to these goals remains the ability of the virus to become resistant, necessitating the development of ever more sophisticated methods to target replication and prevent emergence of resistant strains. The recent use of inhibitors of HIV-1 IN for treating HIV-1 infection has proven to be very beneficial in this regard, with three HIV integrase strand transfer inhibitors (INSTIs) (raltegravir (RAL), elvitegravir (EVG) and dolutegravir) in clinical use. INSTI-based regimens are favoured for ART-naïve HIV-infected persons, based on improved tolerability and side effect profiles, better drug–drug interaction profile and high genetic barrier to resistance^3^. Despite this, resistance has emerged even to these drugs and other methods to target HIV IN such as targeting multimerization of the enzyme or cellular proteins required for IN function present attractive new avenues.

Current anti-HIV treatments require daily administration of a cocktail of drugs and compliance in this is a key feature of failure^4^. Further any new drugs that target HIV proteins, such as HIV IN binders, will also require long term drug regimes. A further critical point in HIV treatment is the latency of the virus in leucocyte pools that can re-emerge in an unpredictable way. The use of biological reagents against infections has been proposed and in particular intracellular antibody fragments^5^. This concept developed into the idea of intracellular immunization^6,7^ where intracellular antibody fragments delivered into cells could interfere with specific disease protein functions^7^. In this respect, the LEDGF-HIV IN interaction represents an attractive therapeutic target^8^ since this mediates viral integration into transcriptional active regions^9^ thereby allowing maintenance of latent HIV for subsequent rounds of viral production^8–10^. The development of small molecule inhibitors of LEDGF and HIV IN for clinical use is in progress^11,12^ but are still at the early stages^13^ and the required potency of compounds that will interfere with this PPI may not be readily achieved as PPIs are hard to block by small compounds^14^. Peptides that inhibit HIV replication have been developed^15^ as an alternative to compounds. Intracellular single domain antibodies offer a further alternative to small molecules for blocking PPIs in cells since these can readily be selected from diverse libraries and bind to specific target antigens within cells with high affinity.

We have expanded on the concept of intracellular immunization by targeting LEDGF/PSIP1 rather than targeting the HIV integrase itself. Our data have been obtained using an intracellular single domain antibody (iDAb), identified by intracellular antibody capture (IAC) which is an in-cell selection method allowing scFv or iDAbs to be identified from diverse intracellular antibody libraries^16^. We show that a T cell line, constitutively expressing an anti-LEDGF/PSIP1 intracellular single domain antibody (iDAb), has a marked reduction in HIV p24 production in HIV-1 infected cells, reflecting interference with infected virus replication.

## Results

### Isolation of a heavy chain variable region single domain antibody binding to LEDGF IBD

We have used IAC technology^16^ to isolate a heavy chain variable region single domain binding to the IBD of the chromatin binding factor LEDGF. This technology uses *in vivo* (in yeast) screening with a two-hybrid strategy to probe diverse naïve human VH iDAb libraries. These libraries comprise a VH3 subgroup framework sequence and are randomized, by a PCR method, at the complementary determining regions (CDR) 3 giving individual libraries with the randomized CDR3 length of between 2 and 15 amino acids. Libraries with different lengths of CDR3 were screened with the IBD bait and clones that grew in the absence of histidine, due to interaction of the IBD bait and a VH-VP16 prey segment permitting transcription of the *HIS3* gene, were selected. Three clones were characterized further (herein called VH59, VH62 and VH65) originating from the libraries with the longest CDR3 (14 or 15 amino acids) and one clone, designated VH59, that originates from the library with CDR3 length 14, was selected on tryptophan, leucine and histidine knockout (WLH-minus) plates at the highest concentration of 30mM 3-amino-1,2,4 triazole (3-AT). The ability of VH59 to bind LEDGF IBD was also confirmed using the yeast system by comparing its binding to both full length LEDGF and the IBD as well as comparing VH62 and VH65 binding properties (Supplementary Figure S1 **)**. VH59 binds to both baits, unlike VH62 and VH65 (Supplementary Figure S1B, grid sectors 2, 3, 5, 7, 8 and 10). VH576, an anti-LMO2 iDAb, and Y6, an anti-Ras iDAb were used as negative controls. VH576-LMO2 and Y6-KRAS pairs were used as positive controls. VH59 was taken forward for detailed study.

**Figure 1:**
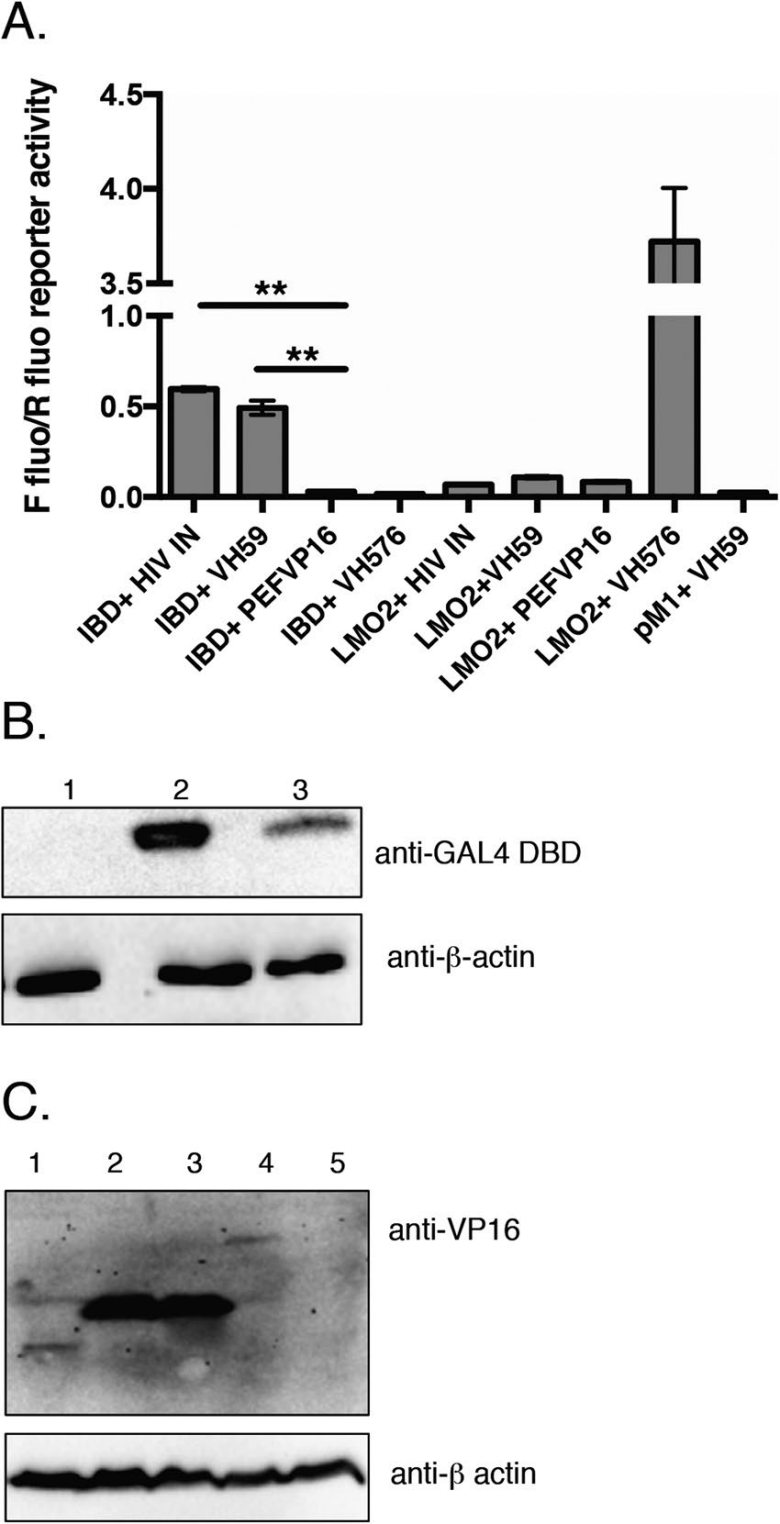
Interaction of LEGDF IBD and anti-IBD VH59 in mammalian cells. The interaction of the LEGDF IBD with the iDab VH59 was tested by mammalian two-hybrid analysis using a luciferase reporter system in CHO cells. LEDGF IBD or LMO2 control baits were expressed as fusions with GAL4 DBD (pM1 vector) and various VH iDAbs or HIV IN were expressed as prey fusions with the VP16 activation domain (pEF-VP16 vector). **(A)** Luciferase reporter activity in transfected CHO cells. Forty-eight hours after transfection, cells were lysed and luciferase activities were measured. Firefly/Renilla luciferase activity was calculated for each transformation pair (**IBD and HIV IN or iDAb VH59 interaction p-value < 0.01, n = 3). LMO2 bait and the anti-LMO2 VH576 were included as a positive interaction controls. (**B)** Western blotting using anti-GAL4 DBD antibody (upper panel) with protein extracts of mock transfected cells (lane 1), CHO cells transfected with pM1-IBD (lane 2) or pM1-LMO2 (lane 3); Protein levels were normalized by probing with an anti-β-actin antibody (lower panel). (**C)** Western blotting using anti-VP16 antibody (upper panel) with protein extracts of CHO cells transfected with pEFVP16 (lane 1); pEFVP16-VH59 (lane2); pEFVP16-VH576 (lane 3); pEFVP16-HIV IN (lane 4); mock transfection (lane 5). Protein levels were normalized by probing with an anti-β-actin antibody (lower panel).

Yeast expressing the IBD and VH59 sustain growth at 30 mM 3-AT, reflecting a high interaction affinity (Supplementary Figure S1C). This feature generally signifies sufficient affinity to support interaction in the more stringent setting of mammalian cell cytoplasm. CHO cells were transfected with mammalian versions of the two hybrid expression vectors, encoding a GAL4 DNA binding domain (DBD)-IBD fusion and a variety of VH domains fused to the VP16 activation domain (Fig. 1A) with luciferase reporter plasmids. A GAL4-DBD-LMO2 bait was used as a negative control and HIV IN was used as a known partner of the LEDGF IBD domain. VH59 showed similar luciferase activity to HIV integrase when co-transfected with LEDGF IBD bait whereas neither prey protein showed luciferase activity with non-relevant bait LMO2 **(**Fig. 1A **)**. Western analysis confirmed the expression of IBD and LMO2 baits using an anti-Gal4 DBD antibody and the expression of prey proteins VH59, VH576 and HIV integrase was confirmed by Western blotting with anti-VP16 antibody **(**Fig. 1B,C **)**. Comparisons of derived protein sequences of VH59 with previously identified iDAbs (anti-LMO2 VH576 and anti-RAS VHY6) are shown in Supplementary Figure S2A and the VH CDR3 is boxed. An alignment of the CDR3 regions of VH59, VH62 and VH65 proteins is shown in Supplementary Figure S2B and the 14 amino acid segment of VH CDR3 that was randomized in the original iDAb library is overlined.

### Structural analysis of the LEDGF IBD-VH59 complex

We determined the crystal structure of the IBD-VH59 complex to obtain detailed information on the mode of binding of VH59 to IBD. The structure was determined at a resolution of 1.7Å by molecular replacement (Supplementary Table 1). We used the structure of the single domain VH anti-RAS intracellular antibody, determined in our previous study (PDB: 2UZI), and the IBD domain crystal structure from its complex with catalytic core domain of HIV-1 integrase (CCD) of HIV IN (PDB: 2B4J) as the search models. Good electron density allowed all four chains of the two independent VH59-IBD complexes, chains H/D and A/E, in the asymmetric unit to be built. The final refined model includes all amino acid residues (1–126) for both VH59 chains, and 347–425, 345–424 for IBD respectively. The full IBD construct encoded residues 345–431 (345–346 were introduced by the cloning procedure), and 14 residues are therefore disordered and invisible in the electron density maps. The intra-domain disulphide bridges are formed in only 30% and 60% of the two independent VH59 molecules respectively, presumably reflecting the intracellular reducing environment where the original selection was carried out and the complex was expressed, and leading to further minor disorder in the structure. The structure was refined to an R factor of 0.187, with an R_free_ of 0.251 (Supplementary Table 1). The R factor difference is higher than expected at this resolution, probably due to the missing IBD termini and disorder around the VH59 disulphide bridges.

**Figure 2:**
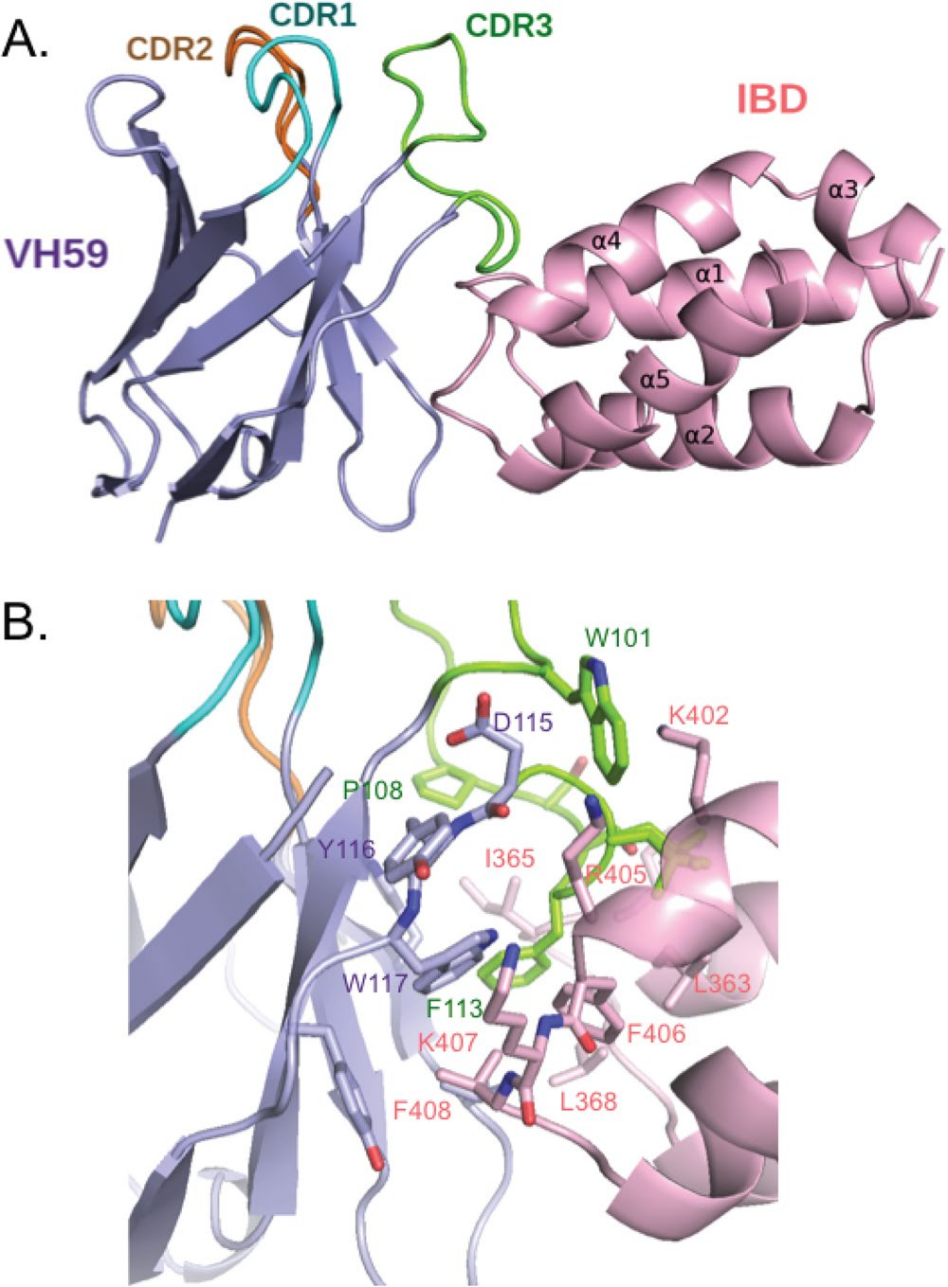
Structure of the LEDGF IBD and iDAb VH59 complex. The crystal structure of the complex between the IBD of LEDGF and the single domain antibody VH59. Panel A: Overall structure of the IBD-VH59 heterodimer: the LEDGF IBD subunit in pink, VH59 in blue. Panel B: The interface in IBD-VH59 heterodimer showing key interacting amino acids; the CDR1 of VH59 is shown in cyan, CDR2 in orange and CDR3 in green.

The two complexes are essentially identical, and one of these (chains H and D) is shown in Fig. 2A. There are, however, some differences between the two VH59 chains in the region of CDR3 not in contact with IBD, and some surface loops, resulting from close contacts with neighbouring molecules in the crystal lattice, but these do not affect the VH59-IBD interface. The LEGDF IBD domain is very compact and contains five helices, while VH59 has an classical immunoglobulin β-sandwich fold acting as a scaffold for the three CDRs (or hypervariable loop regions), that normally mediate interactions with target antigens. Superposition of the IBD on the solution structure of IBD (PDB: 1Z9E), determined by NMR, and those from the other IBD complexes used as molecular replacement search models, showed that VH binding does not affect the overall conformation of the LEDGF IBD (Supplementary Figure S3 **)**.

**Figure 3:**
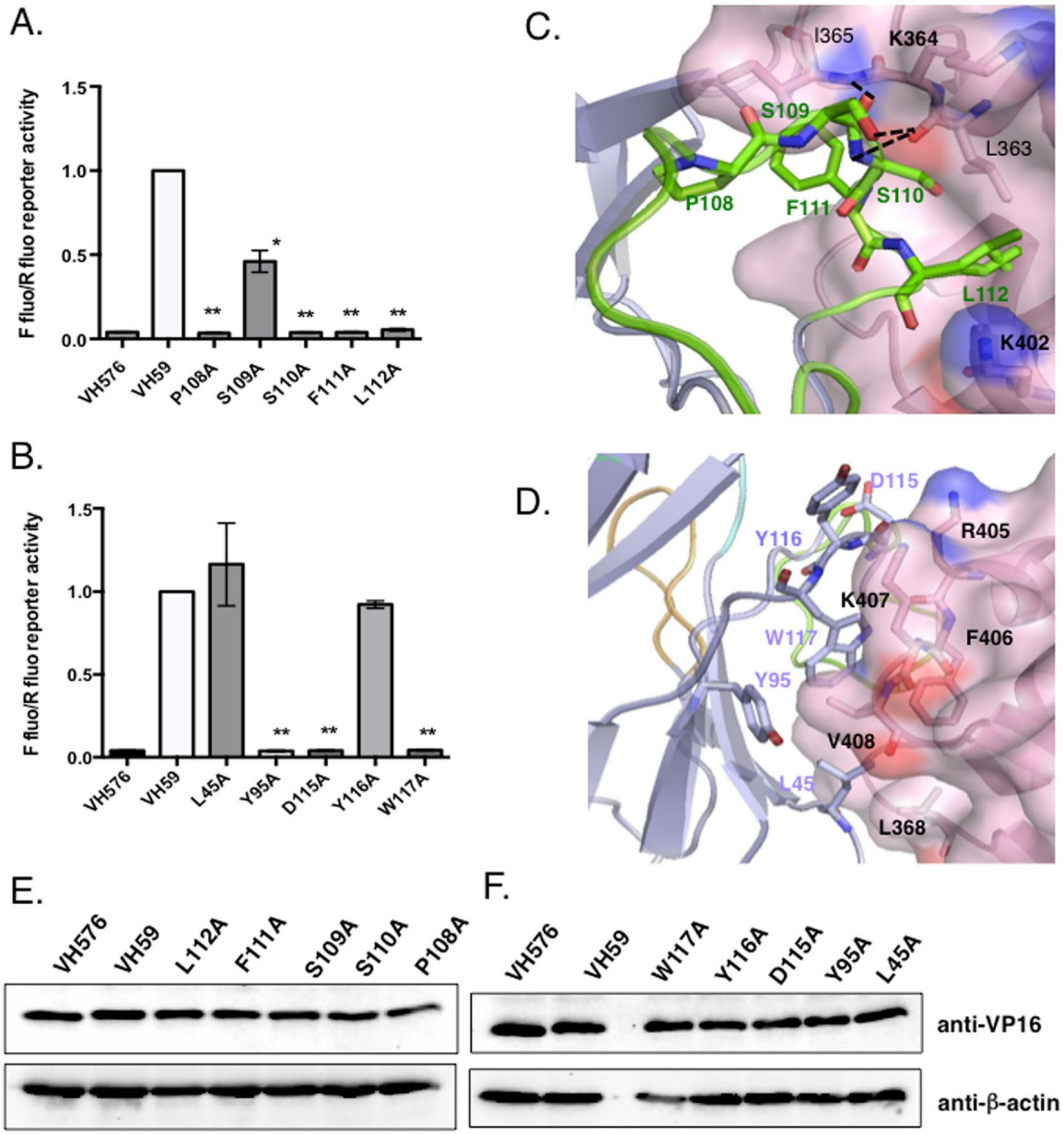
Anchor amino acids in iDAb VH59 determined by alanine scanning. Key amino acids of iDAb VH59 CDR3 and framework region were mutated to alanine using site-directed mutagenesis. pEFVP16-VH59 or mutant VH59 were co-transfected with pM1-LEDGF IBD plasmid into CHO cells. After forty-eight hours of transfection, cells were lysed and luciferase activity was measured. Firefly and Renilla luciferase activities were determined for each VH59 CDR3 mutation **(**panel A**)** or framework mutation **(**panel B**)** p-values are shown as *p < 0.05, ** p < 0.01, n = 3. Key amino acids of VH59 CDR3 **(**panel C**)** and framework region **(**panel D**)** binding to the IBD are indicated in the crystal structure. Western blotting was used to determine expression of VH59 or mutant VH59 fusion proteins with anti-VP16 antibody **(**panels E and F**)** ^41,42^.

The binding site for VH59 on the IBD surface is mainly formed by the two loops that connect helices 1 and 2 (loop1–2 amino acids 364–368) and helices 4 and 5 (loop4–5 amino acids 405–408) of IBD (Fig. 2), with additional contacts to Leu363 and Lys402 at the C-termini of helices 1 and 4, respectively. The binding site on VH59 does not correspond to a normal antibody combining site, with IBD binding to one side of the β-sandwich, rather than interacting with the three CDR at the end of the domain. Only the CDR3 of the iDAb makes contact with IBD, unlike many antibody-antigen interactions where two or three CDR of each domain are involved. The C-terminal region of the long CDR3 turns away from the other CDRs to form a loop (amino acids 108–114) that wedges between the two IBD loops to form the major part of the interface. Additional contacts are made by amino acids 95 and 115–118 either side of CDR3, that formally belong to the framework region, and the interface is completed by contacts from amino acids 45–47 in another framework region, the β-strand preceding CDR2. The overall interaction surface on VH59 is largely hydrophobic and covers an area of 700Å^2^. The IBD surface is mostly complementary, but the complex leaves a central cavity between the molecules, cut off from bulk solvent. This cavity has space for several water molecules, but only one or two, respectively, are ordered in the two independent complexes in the crystal. The IBD-VH59 interactions include four hydrogen bonds, all of which are with CDR3 and mostly via main chain donors and acceptors. (Fig. 3C,D; Supplementary Table 2). The large number, and hydrophobic nature, of the interacting amino acids suggest strong interaction between the proteins, consistent with the observation that IBD-VH59 elutes as single peak on size exclusion chromatography (Supplementary Figure S4).

The key interacting amino acids of VH59 site identified in the crystal structure of the complex were confirmed by mutagenesis. Single mutations of VH59 CDR3 amino acids were made and cloned into pEF-VP16 (Fig. 3, Supplementary Figure S2). These were co-transfected into CHO cells with a pM1 clone expressing the GAL4-DBD-IBD and both a luciferase and Renilla reporter plasmid. The binding site on the VH59 is mainly formed by amino acids with large hydrophobic side chains and mutating those to amino acids with a short aliphatic side chains would be expected to lead to decreased interaction, loss of stereo-complementarity and reduction of the interface area. Indeed, amino acid mutations P108A, Y95A, F111A, L112A and W117A significantly reduced the interaction of VH59 with the IBD (Fig. 3A,B). P108 makes no direct contacts to IBD, but the conformational restriction imposed by proline probably helps stabilize the observed CDR3 loop conformation. Its replacement by alanine could alter the conformation, and the additional flexibility could increase the entropic barrier to binding. The impact of additional direct contacts made to the backbone of the protein and H-bond mediated contacts was confirmed by the IBD mutants S109A, S110A and D115A as they also showed reduced interaction with VH59. This observation underlines the importance of a local conformation for specific recognition between the two proteins. Expression of all the mutated VH59 proteins is comparable to un-mutated VH59 (Fig. 3E,F).

We have measured the Kd using SPR and obtained a value of around 1 μM. We have assessed this with scFv and Dab using biotinylated IBD on the SPR chip and with a second protein preparation of scFv using GST-IBD on the SPR chip (Supplementary Figure S5). We believe that reflects the difference in protein structure and flexibility between periplasmic expressed recombinant scFv, which will have S-S bonds^17^, and the intracellular expressed protein, which will not. The structural analysis of VH in the co-expressed IBD-VH shows a framework interaction of the antibody fragment with IBD, and only partial oxidation of the S-S bridges. The restraints placed on the VH structure by S-S bridges are likely to reduce its affinity for IBD compared to the reduced form, consistent with oxidation being incomplete in the crystal structure despite exposure to oxidising conditions during purification and crystallization. This would also be true for the sample used for SPR-based Kd measurement, so this would represent a lower limit for affinity.

### VH59 and HIV IN catalytic core domain share same binding site on the IBD

**Figure 4:**
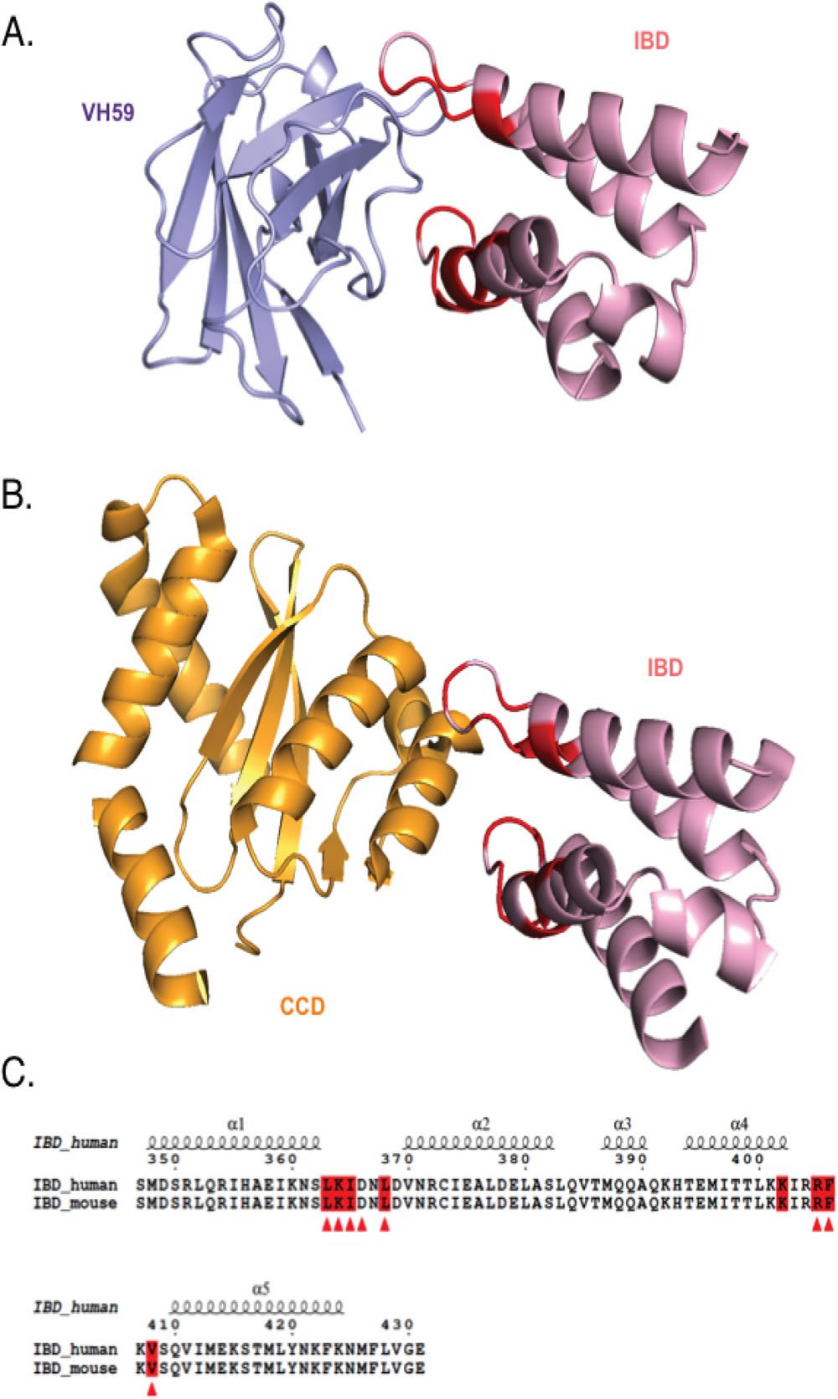
Comparison of LEDGF IBD binding modes with iDAb VH59 and HIV integrase. The crystal structure of the VH59-LEDGF IBD complex determined in this paper is shown in panel A while panel B shows the HIV integrase-LEDGF IBD complex crystal structure (PDB: 2B4J). Panel C shows the co-incidence of amino acids mediating VH59 binding (highlighted in red) and the binding sites of HIV IN (marked with arrows) in sequence alignment, using ESPript 3.0^43^ of mouse and human LEDGF IBD domains indicating the five helical regions.

In order to determine whether VH59 interferes with the binding of partner proteins to IBD, we have made structural comparisons to other known IBD complexes^18^. Alignment of IBD-VH59 and IBD-HIV IN catalytic core domain (CCD) structures (PDB: 2B4J) revealed a significant overlap of binding sites of VH59 and HIV-1 integrase CCD proteins on the IBD surface (Fig. 4). The VH59 binding site covers a broad area on the IBD protein surface that encompasses the whole CCD binding site (Fig. 4A) and extends further to the neighboring area (the overall buried surface for IBD-VH59 is 1391 Å^2^ compared to 1280 Å^2^ for IBD-CCD (Fig. 4B). Indeed, binding of VH59 and CCD are very similar as most of IBD-IN CCD contacts are made to the same amino acids of IBD as those of IBD-VH59 (Fig. 4C highlights these common amino acids in a comparison of human and mouse IBD; Supplementary Table 2).

**Figure 5:**
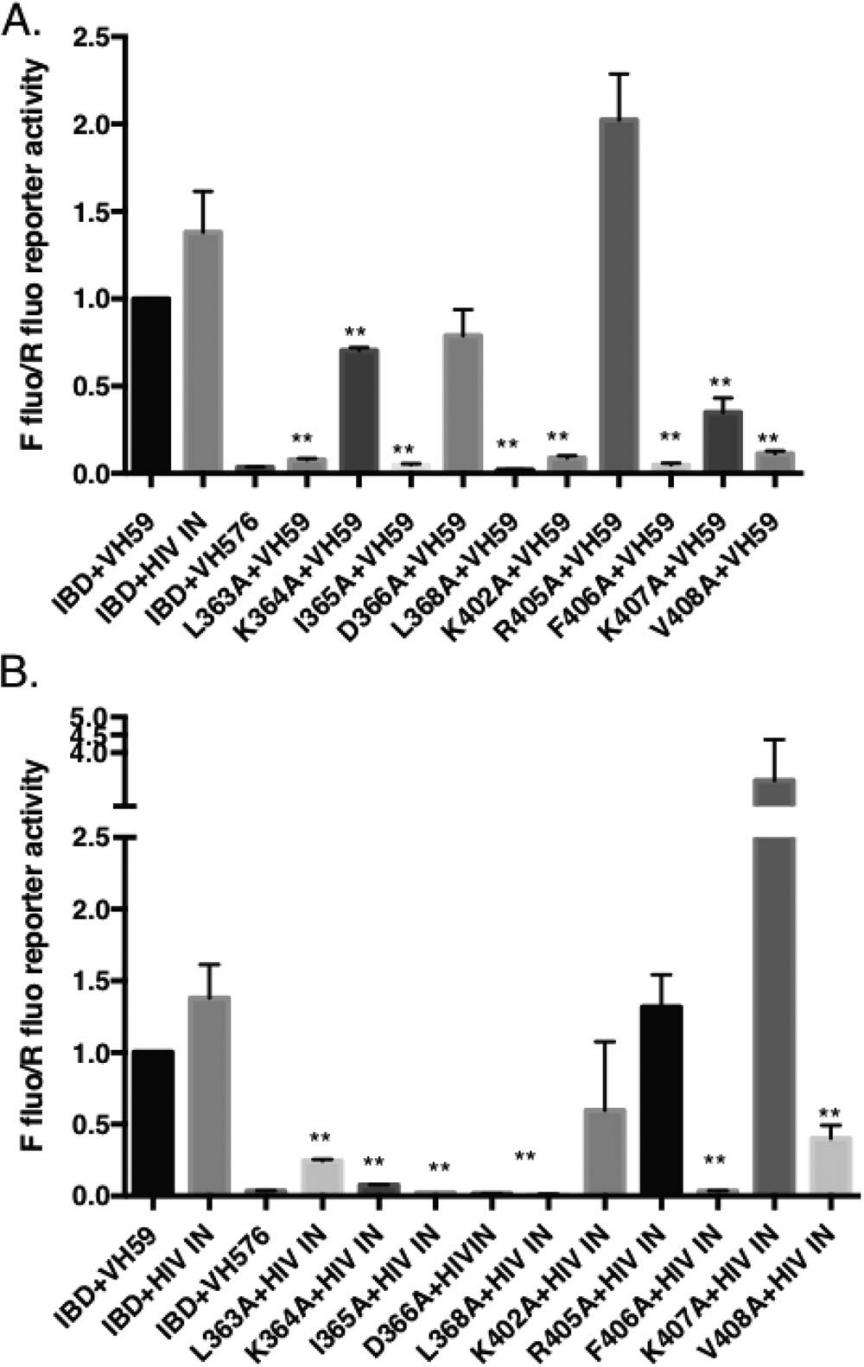
Interaction of mutant LEGDF IBD with VH59 and with HIV IN. Key amino acids of the LEDGF IBD were mutated to alanine using mutagenesis. Expression vectors encoding the mutant proteins were co-transfected in CHO cells with either an expression vector encoding VH59 (panel A) or HIV IN (panel B) together with a Firefly luciferase reporter plasmid and Renilla luciferase expression plasmid for normalization. Forty-eight hours after transfection, cells were lysed and luciferase activities were measured. The interaction of the IBD with wild-type VH59 or HIV IN were used as positive controls and between the IBD and an anti-LMO2 VH (VH576) as a negative control.

The structural alignment of IBD-VH59 and IBD-IN allowed identification of the amino acids on the surface of IBD that interact with both to HIV IN and VH59. As expected, mutations of these amino acids (L363A, I365A, L368A, F406A and V408A) resulted in a significant decrease in the interaction of the IBD with both VH59 (Fig. 5A) and IN-CCD (Fig. 5B).

### VH59 inhibits HIV replication of CD4 T cells by competing HIV integrase binding to LEDGF IBD

**Figure 6:**
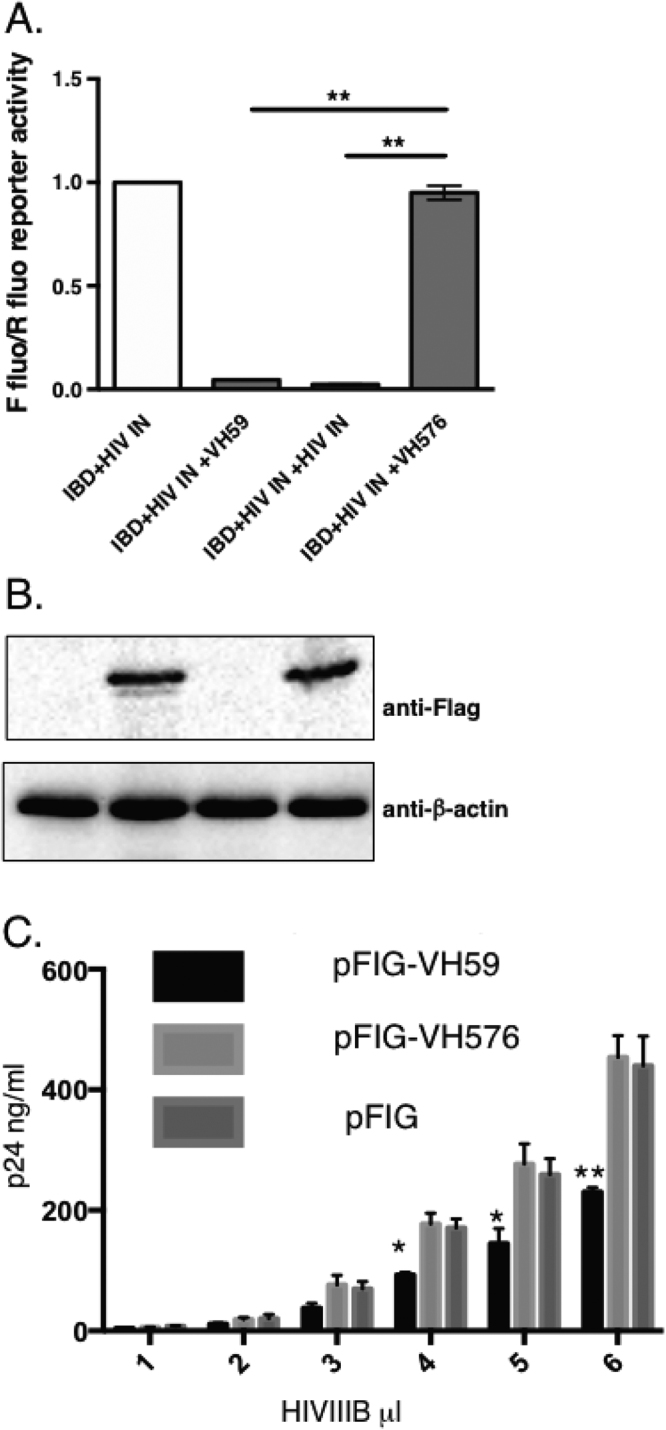
VH59 disrupts the interaction of LEGDF IBD with HIV IN in Jurkat T cell inhibiting the production of viral capsid protein p24. A mammalian LEDGF IBD bait vector (in pM1 to make a GAL2 DBD-IBD fusion) was co-transfected into CHO cells with the prey vector expressing HIV IN (in pEF-VP16 to make an integrase-VP16 activation domain fusion) plus the Firefly and Renilla luciferase reporter plasmids either alone or with an expression vector expressing VH59, HIV IN or an anti-LMO2 VH576. Forty-eight hours after transfection, cells were lysed and luciferase activity was measured. Firefly /Renilla luciferase activity was calculated for each transformation pair (** P < 0.01, n = 3) (panel A). The VH protein levels were estimated using an anti-FLAG tag antibody and normalized using an anti-β-actin antibody (panel B). Jurkat cells were infected with a lentivirus expressing the anti-IBD iDAb VH59, a lentivirus expressing anti-LMO2 VH576 or lentiviral vector alone. Transduced cells were sorted by flow cytometry detection of GFP expression. These cells were infected with escalating doses of HIV IIIB virus and after 5 days, supernatants were collected for p24 protein expression determination by ELISA (Panel C).

The co-incident binding of VH59 to the LEDGF protein at the same amino acids as the HIV IN suggests that VH59 could be a biological inhibitor of HIV by interfering with HIV genome integration. A mammalian three hybrid system was used to assess whether VH59 could compete with the binding of HIV integrase to LEDGF IBD. The interaction of HIV IN-VP16 and the DBD-IBD was assayed in the CHO-luciferase reporter assay (Fig. 6A) and this interaction was not affected by co-expression of a non-relevant VH (anti-LMO2 VH576). Conversely, co-expressing the HIV IN-VP16 and Gal4-IBD proteins with either free VH59 or HIV IN resulted in about 90% inhibition of luciferase production due to competition. Western blotting confirmed the expression of VH59 and VH576 proteins in this assay **(**Fig. 6B).

In HIV-1 infections, CD4-positive T cells express IN that interacts with LEDGF for nuclear import and chromatin tethering of the pre-integration complex, facilitating integration of the viral DNA into the host genome^19^. Since VH59 acts as a competitor of LEGDF-HIV IN interaction, we tested if this competitive binding ability would have an inhibitory effect on HIV replication. We used an HIV infection assay in which VH59, or anti-LMO2 VH576 as a control, was expressed from a lentivirus vector in the CD4-expressing Jurkat cells and effects on HIV replication was measured by determining production of the p24 capsid protein **(**Fig. 6C **)**. Increasing levels of HIV virus were used to infect VH59 or VH576 transduced Jurkat cells and p24 expression level was determined after 5 days. We observed that the p24 levels in VH59 expressing cells were significantly lower than the controls at all HIV virus titres tested. In cells infected with the highest viral titre, presence of VH59 suppressed the expression of p24 by 50% compared to p24 in cells expressing the non-relevant VH iDAb or the empty lentiviral vector (**P < 0.01). The inhibitory effect of VH59 on the interaction of LEDGF and HIV IN translates into an inhibitory effect on the production of HIV virus if cells have been pre-loaded with VH59 before HIV exposure. This means that the VH59-expressing cells have effectively undergone intracellular immunization.

## Discussion

### An intracellular domain antibody binding to the HIV IN binding domain of LEDGF

Intracellular antibody capture technology (IAC)^20,16^ was developed to allow selection of antibody fragments from diverse libraries in the reducing environment of cells to allow their binding to intracellular target proteins. It was originally developed with scFv selection^20^ and later using single variable region iDAbs^16^. Diverse libraries of VH segments, based on a human VH3 subgroup sequence, were generated with diversity at the CDR3 region for screening in yeast against antigens of choice^16^. The binding affinities of initial hits can be improved as required by an *in vivo* affinity maturation protocol^21^. The iDAb described here (VH59) was developed using IAC selection of a diverse VH library with CDR3 length 14 amino acids.

Blocking the PPI between LEDGF and HIV IN can affect HIV replication^15^ and small molecule drugs that work on the LEDGF-HIV IN interface could potentially be important for therapy. However, those in development are still not in clinical use owing to of potency issues, probably because the flat and rather featureless interaction surface represents a poor target for small molecules that bind more strongly in clefts. Compounds that bind to either HIV IN^11,13,22^ or to LEDGF/p75^23^ have been isolated and are progressing to clinical use. However, macromolecules (such as iDAbs), that bind either LEDGF or HIV IN, would also have potential for therapeutic application in early HIV infections and implementation should be possible by coupling new genome editing methods with using new delivery methods^24,25^ to allow expression of iDAbs in CD4+ T cells.

Several intracellular antibodies have been described against HIV proteins, including structural proteins, TAT, REV, reverse transcriptase and integrase (reviewed in^26^). In our study, we have selected a heavy chain variable region iDAb, using IAC screening that binds the cellular protein LEDGF and proved to compete with the binding of HIV IN to LEDGF. The crystal structure of the IBD-VH59 complex shows a large area of interaction between the binding partners, as often observed in antibody-antigen complexes, with a particularly high proportion of hydrophobic interactions and few hydrogen bonds. More striking is the observation that IBD is not bound by all three CDR loops, but lies alongside VH59 domain. This unusual antigen binding site is not unprecedented for VH complexes, and is similar to that observed for the complex between the anti-LMO2 VH576 and its LMO2 target^27^, where CDR3 is the only hypervariable loop involved, in addition to contacts within the VH framework amino acids. This is probably the result of the IAC screening procedure, where the VH59 protein sequence was derived from a diverse VH library with 14 amino acids of CDR3 randomized while CDR1 and CDR2 were not^16^. The framework interactions can therefore provide additional possible binding surfaces and compensate to some extent for the lack of CDR1 and CDR2 diversity. In addition, the framework region on the face of the VH59 β-sandwich adjacent to CDR3 is hydrophobic, since this is the surface that would be buried in the interface to VL in a normal antibody. This is, therefore, complementary to the hydrophobic nature of the interaction site on IBD.

### VH59 mimics HIV IN binding sites on LEDGF

Our intracellular antibody binds the cellular protein LEDGF, and the binding site of HIV IN on the IBD of LEDGF has been mapped onto two loops on the surface^28^. The crystal structure indicated that VH59 binds to LEDGF IBD at an almost identical position as the integrase. The conformation of the IBD in the context of the full length LEDGF protein is not known although it must be accessible to integrase and is very likely to be the same as in the LEDGF IBD-IN crystal structure. It is therefore also likely to bind VH59. The crystal structure analysis, and site-directed mutagenesis of VH59 and IBD, show common binding amino acids on LEDGF IBD that interact with VH59 and HIV IN (Fig. 5). This explains why VH59 competes with the binding of HIV integrase with LEDGF. The property of inhibiting LEDGF IBD-IN interaction makes the VH59 iDAb a potential biological inhibitor of HIV infection by interfering with HIV replication.

The probability of resistance development to VH59 should be low, as it targets a cellular protein. I could be wrong but I didn’t think LEDGF had enzymatic activity(?) by contrast with integrase inhibitors. iDAbs have a larger binding surface area unlike HIV integrase inhibitors, such as raltegravir that are also susceptible to mutations, to which the HIV genome is prone^29^. Our data show that binding of an iDAb to the LEDGF surface, rather than the HIV genome-encoded integrase, is effective and this protein is unlikely to acquire mutations in view of its function. While Legdf/*Psip1* gene in mouse knockouts show perinatal lethality^30^, these knock-out mice have normal immune systems and TALEN-based depletion of human CD4+ T cells show no deleterious effects of losing LEDGF^31^. In an *in vivo* implementation of LEDGF-iDAb binding in T cells, this should not produce a phenotype other than preventing HIV productive genome integration.

### The intracellular domain antibody is an inhibitor of HIV

LEDGF is a transcription co-factor that interacts with several proteins, traffics into the nucleus and binds strongly to chromatin (Christ and Debyser, 2013). This protein has functions in MLL-leukaemias mediated by binding of MLL and Menin to IBD helix 5^32^. The interaction of HIV IN with LEDGF is needed for the uptake of the viral pre-integration complex into nuclei of infected cells for chromatin binding and viral genome integration^8^. In view of this, there is great interest in developing inhibitors to block LEDGF and HIV-1 protein interactions for potential therapy of HIV infection. As an alternative to small compounds, we have described a new inhibitor of the interaction of HIV IN and LEDGF with the ability to interfere with the HIV virus replication cycle. This is the first intracellular antibody that binds this cellular protein rather than an HIV encoded protein. Using *in vitro* HIV IIIB infection of CD4 T cells as a model, we show that VH59 inhibits virus production measured by decreased p24 capsid protein levels in the supernatant. Our use of iDAb VH59 shows that by pre-loading target cells with an antigen-specific intracellular single domain antibodies, we can effectively immunize against HIV-1 infection. This type of intracellular immunization has potential for use in treating HIV-1 infected individuals through stem cell genome editing approaches wherein all subsequently progeny CD4+ T cells will express the anti-viral VH and thus would be impervious to continued HIV-1 infection. This would permit healthy T cell re-population. However, for this intracellular immunization technology to become clinically feasible, the uptake of the therapeutic in a cell-specific manner is dependent upon *in vivo* delivery system advances^24^ (although this is also true of other biological therapies currently in development).

### Conclusions and Future Prospective

We show that blocking LEDGF interaction with HIV IN in cells constitutively expressing an intracellular single domain antibody binding to LEDGF interferes with HIV replication. This is distinct from intracellular antibodies targeting viral proteins and small molecule inhibitors of LEDGF:HIV IN and indicates that anti-LEDGF iDAb expression in CD4+ T cells will be without deleterious effects *per se* whilst influencing HIV replicative processes.

One reason why anti-HIV intracellular antibodies have not progressed to clinical use is the difficulty of their delivery to target cells in patients. Our paper shows that adding an intracellular antibody expression gene to the genome gives immunity to these cells. This is ‘intracellular immunization’^7^ as the cell has prior production of an single domain antibody to target a viral antigen. We envisage that cell-specific antibody fragment expression will be possible when the new methods of genome editing (viz. CRISPR/cas9-mediated genome integration) are achieved to specifically express iDAbs in T cells or macrophages (the targets of HIV infection or reservoirs of latent virus). Intracellular immunization has potential for therapeutic application in early HIV infections and implementation should be possible using new delivery technologies that are becoming feasible^24,33,34^. Such a gene editing approach has recently been used in the correction of mutations in human embryos^35^ and promises to provide the means to stably alter cells to express exogenous genes such as those encoding intracellular antibodies. These technologies that will facilitate intracellular immunization will be alternatives to small molecule drugs that may lack potency and to which resistance can emerge.

## Materials and Methods

### Intracellular antibody capture (IAC) selection of VH iDAbs

A construct encoding the LEDGF IBD (amino acids 345–431) was cloned into the yeast bait vector pBTM116 and transfected into L40 yeast. Eleven single domain VH iDAb diverse libraries, with randomized CDR3 loops ranging from 5 to 15 amino-acids^16^ were transfected into the LEDGF IBD yeast strain and clones showing IBD-VH interaction were identified by growth of yeast on plates lacking tryptophan, leucine and histidine^16^. Three VH clones were identified and were selected for study (designated VH59, VH62 and VH65).

### Mammalian two-hybrid and three-hybrid analysis

The LEDGF IBD coding region was cloned into the bait expression vector pM1, and VH59 and VH576 (an anti-LMO2 iDAb) were cloned into the prey expression vector pEF-VP16^16^. For mammalian two hybrid analysis, bait and prey vectors were transfected into CHO cells using lipofectamine 2000 (Invitrogen) together with the Firefly luciferase reporter pG5-Fluc and control Renilla luciferase vector pRL-CMV. Forty-eight hours after transfection, Firefly and Renilla luciferase enzyme activities were measured using the Dual-Luciferase reporter assay system (Promega) according to the manufacturer’s instructions. Data were analyzed using a Lucetta^TM^ Luminometer (Lonza). The relative reporter activity was calculated as a ratio of Firefly luciferase activity/Renilla luciferase activities.

For three-hybrid competitor analysis, competitor cDNA sequences were cloned into the pEF-BOS-cyto vector (Invitrogen) and bait, prey and competitor plasmids were co-transfected into CHO cells together with pG5-Fluc and pRL-CMV reporter vectors. The luciferase activities were measured as for the mammalian two hybrid assay. Statistics were carried out using paired T-test. * indicate P < 0.05, ** indicate P < 0.01.

### LEDGF IBD and VH59 protein co-expression in *E. coli*

LEDGF IBD and VH59 iDAb were co-expressed in *E. coli* C41(DE3) cells. The LEDGF IBD coding region (amino acids 347–431) and the VH59 sequence were cloned into the pRK-172 vector in-frame with an N-terminal 6 × his-tag and a TEV protease recognition site with the two cistrons linked by an internal ribosome binding site (RBS). The plasmid containing IBD-VH59 sequences was transformed into C41(DE3) cells and grown in 50 ml LB medium with 100 μg/ml ampicillin for 24 hours, and subsequently added to 1 L LB medium. Protein expression was induced at OD = 0.2 by addition of 0.5 mM IPTG and grown at 37 °C for 5 hours. Bacteria were collected and sonicated in 20 mM Tris-HCl pH8, 250 mM NaCl, 20 mM imidazole, 0.5% NP40, 5 mM β-mercaptoethanol and EDTA-free protease inhibitor cocktail (Roche Diagnostics, Mannheim, Germany). Proteins were purified using nickel agarose beads (Invitrogen) and bound proteins were eluted in 20 mM Tris-HCl pH 8, 250 mM NaCl, 300 mM imidazole, EDTA-free protease inhibitor cocktail. HIS-tagged TEV protease (1.5 mg/ml) was added at a ratio of 1:100 and dialysed against 20 mM Tris-HCl pH 8, 250 mM NaCl. Protease and free HIS-tag were removed using nickel agarose beads (Invitrogen) and unbound proteins were eluted in 20 mM Tris-HCl pH 8, 250 mM NaCl, 300 mM imidazole, EDTA-free protease inhibitor cocktail. The final samples were concentrated using Amicon Ultra-15 centrifugal filter devices with a 10 kDa cut off (Millipore, MA, USA). The proteins were further purified by gel filtration on a HiLoad Superdex 75 10/300GL column (GE Healthcare, Uppsala, Sweden) in a buffer containing 10 mM Tris-HCl, pH 8, 150 mM NaCl at a flow rate of 1 ml/min. Fractions corresponding to the protein complex were pooled and concentrated to 6 mg/ml for crystallization trials. Protein concentrations were determined by Bradford assays. Protein purity was analyzed by SDS-PAGE stained with Coomassie Brilliant Blue.

### VH59-IBD crystallization and structural determination

Crystallization experiments were carried out in 96-well Greiner plates using 200 nanolitre droplets. Crystallization plates were stored at 4 °C and examined at regular intervals. Crystals of the IBD-VH59 complex suitable for X-ray diffraction grew in 25.0% w/v Polyethylene Glycol 3350, 0.1 M Tris pH 8.5. Crystals were cryo-protected with Paratone and flash-cooled in liquid nitrogen. X-ray diffraction data were collected at station I04-1 of the Diamond Light Source (DLS, Oxfordshire). A total of 1800 diffraction images were collected, each with an oscillation range of 0.2°. The IBD-VH59 crystals belong to space group P1, with unit cell dimensions a = 35.1, b = 41.4, c = 58.7 Å, α = 104.0, β = 96.1, γ = 100.5°. The structure was solved by molecular replacement. The asymmetric unit contains two copies each of IBD and VH proteins. An initial search was done in PHASER and included two molecules of VH^36^. Additional searches performed in MolRep allowed correct localization of the two missing molecules of IBD^37^. Model building was carried out using Coot^38^ and refinement was done with REFMAC^39^. Data collection and refinement statistics are shown in Supplementary Table 1.

### SDS-PAGE and Western analysis

CHO cells transfected with different bait and prey vectors were lysed in 2% SDS/PBS, and boiled in sample buffer (100 mM Tris-HCl pH 6.8, 10% (v/v) glycerol, 2% (w/v) SDS and 2% (v/v) β-mercaptoethanol). 30μg of protein were fractionated on 12% denaturing acrylamide gels. For Western blotting, proteins were transferred to polyvinylidene difluoride (PVDF) membranes, blocked with 5% non-fat milk and incubated with either primary antibody mouse anti-VP16, mouse anti-Gal4 DBD (Santa Cruz Biotech) or mouse anti-β-actin (Sigma-Aldrich) (the latter was for loading control). The membranes were washed three times with 10 mM Tris, pH 7.5, 50 mM NaCl and 0.1% (v/v) Tween-20 and incubated with horseradish peroxidase-labeled anti-mouse antibody (Sigma-Aldrich). A ChemiDoc MP enhanced chemiluminescence imaging system (GE Healthcare, Uppsala, Sweden) was used for data collection.

### Preparation of GST-IBD and scFv-59

GST-IBD: cDNA encoding the integrase binding domain, residues 345–431 of LEDGF/p75, was cloned into the pGEX-2T vector in-frame with the C-terminal Glutathione-S transferase (GST). The pGEX-GST-IBD plasmid was transformed into *E. coli* C41 (DE3) cells for protein expression. Bacterial cells were cultured in LB media containing the appropriate antibiotics at 37 °C, 225 rpm until an OD_600_ of 0.6 was reached. Protein expression was induced by the addition of 0.1 mM IPTG (isopropyl 1-thio-beta-D-galactopyranosid) and the cells were incubated overnight at 16 °C, 225 rpm. Cells were harvested by centrifugation and the pellet was resuspended in lysis buffer (10 mM Na_2_HPO_4_, 1.8 mM KH_2_PO_4_, 2.7 mM KCl, 140 mM NaCl, pH 7.3) containing EDTA-free protease inhibitor cocktail tablets (Roche, Germany). Cells were lysed using a cell disruptor (Constant Systems Ltd., UK) at 25,000 psi, 4 °C and the sample was clarified by centrifugation at 50,000 × g for 1 hour. The cell lysate was incubated with glutathione Sepharose 4B (GE Healthcare, UK) for 2 hours at 4 °C. Soluble GST-IBD was eluted using 50 mM Tris, 10 mM reduced glutathione (GSH), 0.1 ZnSO_4_, pH 8.0. The concentrated protein sample was further purified by gel filtration using a HiLoad 16/600 Superdex 75 column (GE Healthcare, UK) in a buffer containing 20 mM Tris, 150 mM NaCl, 1 mM DTT, pH 8.0. The purified GST-IBD protein was concentrated to 0.5 mg/ml for SPR experiments.

VH59-scFv: was cloned into the pSANG10 vector in-frame with C-terminal 6xHis and FLAG tags. Plasmid DNA was transformed into *E. coli* C41 (DE3) cells for protein expression. A single colony was used to inoculate 50 ml of 2xTY containing 2% glucose and 50 ug/ml kanamycin, which was grown overnight at 30 °C, 225 rpm. The overnight seed culture was diluted 1:100 in 4 × 1 L of 2xTY containing 0.1% glucose and 50 ug/ml kanamycin. The cultures were grown at 37 °C, 225 rpm until an OD_600_ of 0.8 was reached. Protein expression was induced by the addition of 1 mM IPTG (isopropyl 1-thio-beta-D-galactopyranosid) and the cells were incubated overnight at 30 °C, 225 rpm. Cells were harvested by centrifugation and protein extracted by periplasmic osmotic shock. The cell pellets were resuspended in TES buffer (50 mM Tris, 1 mM EDTA, 20% sucrose, pH 8.0) containing EDTA-free protease inhibitor cocktail tablets (Roche, Germany) and 100 ug/ml lysozyme (50 ml/L culture). Following a 10 minute incubation on ice, the cells were centrifuged at 4000 rpm for 10 min at 4 °C. The supernatant was retained and the pellet was resuspended in an ice-cold 5 mM MgSO_4_ solution containing protease inhibitors and 100 ug/ml lysozyme (50 ml/L culture). Both supernatants were pooled and the mixture were clarified by centrifugation at 11,000 rpm for 45 minutes. The periplasmic extract was incubated with Ni-NTA Agarose (QIAGEN, UK) for 2 hours at 4 °C. The soluble VH59-scFv protein was purified from the beads using 50 mM Tris, 400 mM imidazole, 500 mM NaCl, pH 8.0. The protein was dialysed into PBS, pH 8.0 using SnakeSkin Dialysis Tubing with a MWCO of 3,500 Da (Thermo Fisher Scientific, UK). The VH59-scFv protein was concentrated to 1.5 ml and purified further by gel filtration using a HiLoad 16/600 Superdex 75 column (GE Healthcare, UK) in PBS, pH 8.0. Fractions containing pure VH59- scFv were pooled and concentrated to 0.2 mg/ml for SPR experiments.

### Surface plasmon resonance

A BIAcore T200 (GE Healthcare) was used to measure the affinity of the VH59-scFv binding to GST-IBD. A polyclonal goat anti-GST antibody (GE Healthcare) was immobilised on a CM5 sensor chip (GE Healthcare) by the amine coupling method and the GST-IBD protein was immobilized via the antibody. To immobilize the antibody on CM5 chip, the chip was first activated by injecting 100 µl mixture of 0.2 M EDC (N-ethyl-N-(dimethylaminopropyl) carbodiimide hydrochloride) and 0.05 M NHS (N-hydroxysuccinimide) at 10 µl/min flow rate. 100 µg/ml anti-GST antibody in 10 mM sodium acetate, pH 5.0 was injected at 5 ul/min for 900 sec and immobilized until 9,000~12,000 RU. After immobilization the chip was immediately inactivated by injecting 1 M ethanolamine, pH 8.5 at 10 µl/min for 600 sec. The chip was kept running in the HBS buffer (GE Healthcare) comprising 10 mM HEPES, pH 8.0, 150 mM NaCl. 5 µg/ml recombinant GST and GST-IBD proteins were injected into flow cell Fc1(GST) and Fc2, Fc3 and Fc4 (all GST-IBD). All proteins were trapped through the immobilised anti-GST antibody. The GST-IBD protein was immobilized up to 2,5000 RU in Fc2, 4,000 RU in Fc3 and 9,300 in Fc4 via a customized method using The BIAcore evaluation 2.1 software. The protein was injected at 5 µl/min for sufficient time to reach the desired level of immobilization. Before the scFv dose response experiment was carried out, the running buffer was replaced by 1X PBS pH 8. A dose response experiment was set up with a series of dilutions of the initial concentration for the VH59-scFv (all in PBS pH 8). The dilution series were 6.1, 3.1, 1.53, 0.76, 0.38, 0.19, 0.095, 0.048, 0.024, and 0.0 uM and in triplicate in a polypropylene 96 well plate. The protein was injected at 30 ul for 50 sec at 20 ul/min flow rate over flow cells Fc1, Fc2, Fc3 and Fc4. The corresponding kinetic rate constants, K_on_ and K_off_, were determined using steady-state affinity and the Fit Kinetic analysis of the compounds’ binding affinities, assuming 1:1 ligand-protein stoichiometry.

### Site directed mutagenesis

Primers for amplification of mutant forms of the anti-IBD iDab VH59 or LEDGF IBD were designed using the GeneArt Primer and Construct Design online tool. Template DNA was amplified with KOD polymerase enzyme (Novagen, Nottingham, UK), PCR products were digested by Dpn I at 37^0^C for 1 hour and ligated into respective backbone vector followed by transformation into XL-1 blue *E. coli*. Positive clones were confirmed by DNA sequencing (Source Bioscience, Nottingham, UK).

### Lentivirus infection of Jurkat T cells and measurement of p24 viral capsid protein

Anti-LEDGF VH59 and anti-LMO2 VH576 were cloned into the pFIG lentiviral vector^40^ to yield pFIG-VH59 or pFIG-VH576, co-expressing VH and a green florescence protein (GFP) reporter. These plasmids or pFIG empty vector were co-transfected with the packaging plasmid p8.91 and the envelope plasmid pMD2.G into HEK293TLA cells. The transfected cells were cultured for 2 days and lentivirus particles were harvested from the culture supernatant. Jurkat cell lines expressing VH59 or VH576 were established by infecting cells with lentivirus in the presence of 8μg/ml protamine. Cells were cultured for 3 hours, fresh medium was added and cultured for further 2 days. Transduced cells expressing GFP were separated by flow cytometry. The GFP-positive Jurkat cells expressing VH were seeded at a density of 2.5 × 10^6^ cells/ml in 96-well plates at 40 μl/well. HIV-1 IIIB virus, was added at serial dilutions (6 μl virus equal to MOI 0.1), mixed with cells and cultured for 90 min. Cells were centrifuged and washed to remove free virus prior to re-suspension in RPMI, 10% FCS. Cells were cultured for 5 days prior to collection of supernatant and measurement of p24 gag protein by ELISA (Advanced BioScience Laboratories Inc).

The co-ordinates of the IBD-VH59 complex have been submitted to the Protein Data base and assigned number 5N88.

## Electronic supplementary material

Supplementary information
