# Supplementary material for: Intracellular immunization against HIV infection with an intracellular antibody that mimics HIV integrase binding to the cellular LEDGF protein
Source: Sci Rep. 2017 Dec 4;7:16869. doi: 10.1038/s41598-017-16742-2 (PMC5715112; doi:10.1038/s41598-017-16742-2)
Supplement: Supplementary file 1 — Supplementary information [file 41598_2017_16742_MOESM1_ESM.pdf]

Intracellular immunization against HIV infection with an intracellular antibody that mimics HIV integrase binding to the cellular LEDGF protein

Leyuan Bao, Clare Hannon, Abimael Cruz-Migoni, Denis Ptchelkine, Mei-yi Sun, Ami Miller, Wilawan Bunjobpol, Camilo E. Quevedo, Mariliza Derveni, Jennifer S. Chambers, Alison Simmons, Simon E.V. Phillips, Terence H. Rabbitts

Supplementary Data Contents

Figure S1
Figure S2
Figure S3
Figure S4
Figure S5

Table S1
Table S2

Supplementary Figure 1

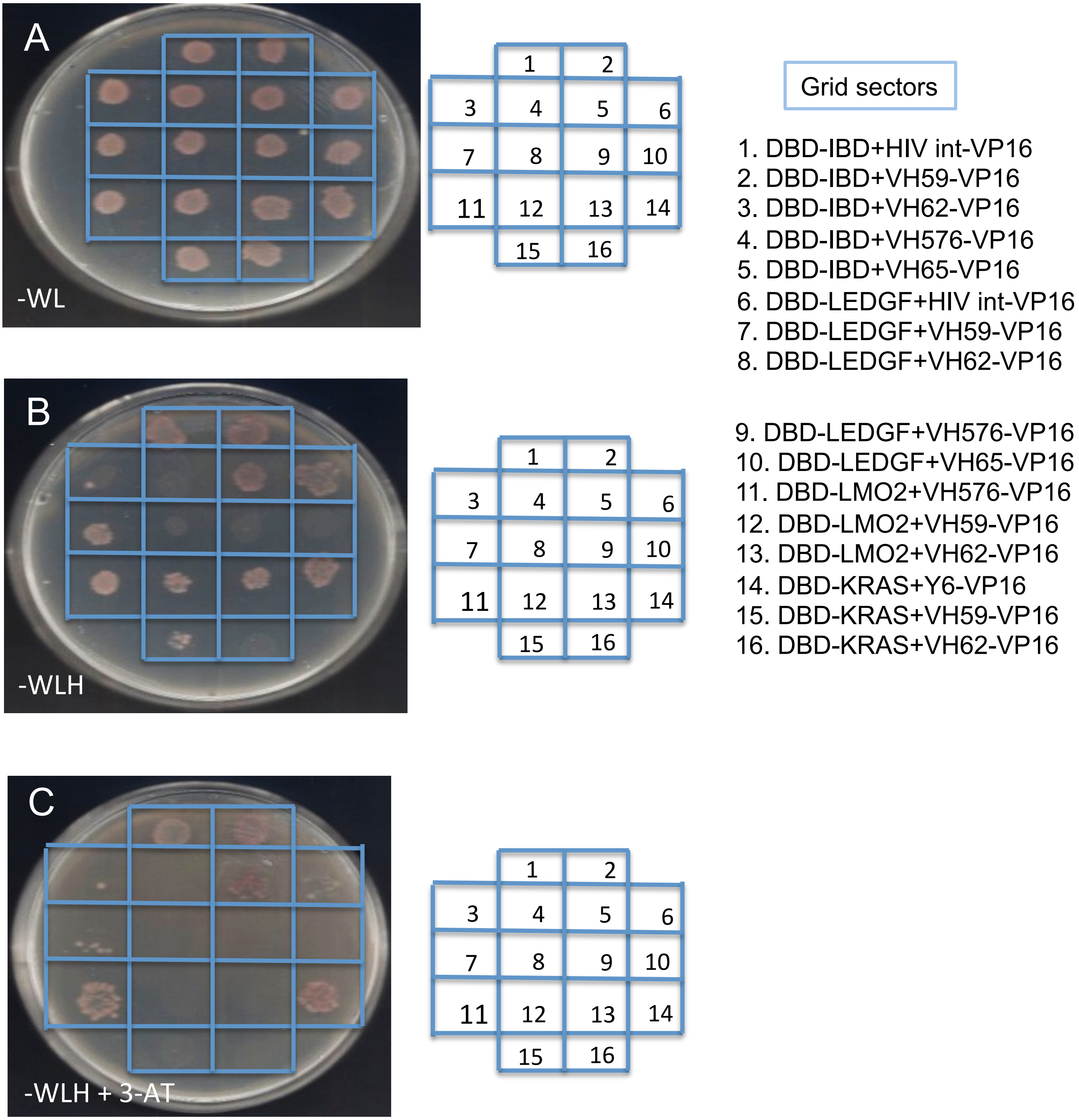

Figure S1. Characterization of VH59 interaction with LEDGF using yeast intracellular antibody capture

Three anti-LEDGF IBD VH single domains, isolated by IAC, in pVP16 were co-transfected with various bait vectors in pBTM116 (as indicated grid sectors) into yeast L40 and clones picked to make a master plate grown in absence of tryptophan (W) and leucine (L) (panel A). These yeast clones were replica plated in the absence of tryptophan, leucine and histidine (H) (panel B) or in the absence of WLH plus 30mM 3-AT (panel C).

The GAL4 DBD fusions indicated are HIV int (HIV integrase), IBD (LEDGF IBD), LEDGF (full length LEDGF), LMO2 or KRASG12V. The VP16 activation domain fusions tested were anti-LEDGF IBD clones 59, 62, 65; anti-LMO2 VH576; anti-RAS VH Y6 and HIV int.

Supplementary Figure 2

A.

```
                                                                   CDR1                   CDR2
VH59_protein_sequence      MAEVQLLESGGGLVQPGGSLRLSCAASGFTFSTFSMNWVRQAPGKGLEWVSYISRTSKTI
VH576_protein_sequence     MAEVQLLESGGGLVQPGGSLRLSCAASGFSFSHSPMNWVRQAPGKGLEWVSYISYNSSSI
Y6_protein_sequence        MAEVQLLESGGGLVQPGGSLRLSCAASGFTFSTFSMNWVRQAPGKGLEWVSYISRTSKTI
                           *****************************:**   ***************** .*.:*

VH59_protein_sequence      YYADSVKGRFTISRDNSKNTLYLQMNSLRAEDTAVYYCARGGWALGDEIPSSFLEFDYWG
VH576_protein_sequence     YYADSVKGRFTISRDNSKNTLYLQMNSLRAEDTAVYYCARGLT---ESLELTADWFDYWG
Y6_protein_sequence        YYADSVKGRFTISRDNSKNTLYLQMNSLRAEDTAVYYCARGRF------------FDYWG
                           *****************************************              *****
                                                                        CDR3

VH59_protein_sequence      QGTLVTVSS
VH576_protein_sequence     QGTLVTVSS
Y6_protein_sequence        QGTLVTVSS
                           *********
```

B.

```
VH59     ARGGWALGDEIPSS-FLEF
VH62     ARGNVSLRCEPLRDWCPGF
VH65     ARGGESMNGFLPAGYWFQF
         ***   ::          *
```

Figure S2. Comparison of VH59 protein sequence with previously identified iDAbs

A. The derived proteins sequence of anti-LEDGF IBD VH59, anti-LMO2 VH576 and anti-RAS VHY6 protein sequences were aligned using Clustal Omega online software. CDR1, CDR2 and CDR3 region are indicated according to IMGT nomenclature http://www.imgt.org/ .

B. Comparison of the CDR sequences of anti-LEDGF IBD VH clones 59, 62 and 65. The three clones were derived from a diverse VH library with 14 amino acids of CDR3 randomized[1], although clone VH59 lacks one residue indicated by -. The CDR1 and CDR2 sequences of all three clones are identical.

The 14 amino acids of CDR3 that were randomized in the preparation of the diverse VH library [1] are overlined.

Reference

1. Tanaka, T. & Rabbitts, T. H. Protocol for the selection of single-domain antibody fragments by third generation intracellular antibody capture. *Nature Protocols* **5**, 67-92 (2010)

Supplementary Figure 3

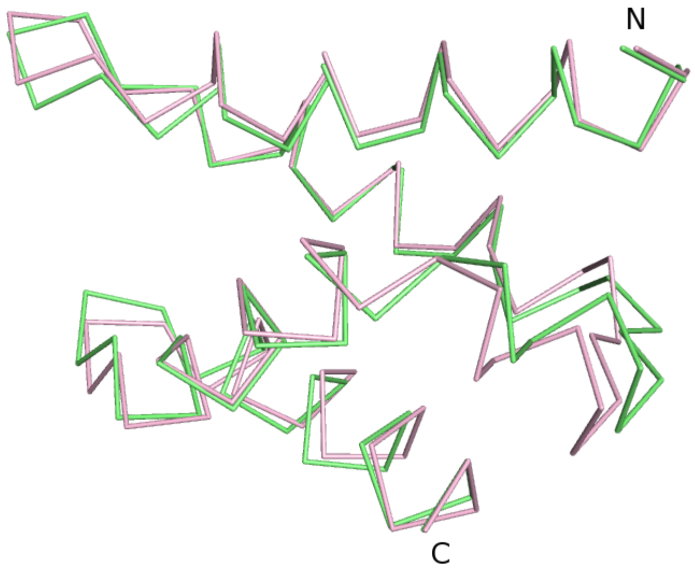

Figure S3. Comparison of the structure of LEDGF IBD from the IBD-VH59 complex with LEDGF IBD alone

Super-imposition of the C-alpha traces of the IBD crystal structure when bound in complex with CCD (2b4j, green) and of the IBD from the IBD-VH59 complex (magenta).

Supplementary Figure 4

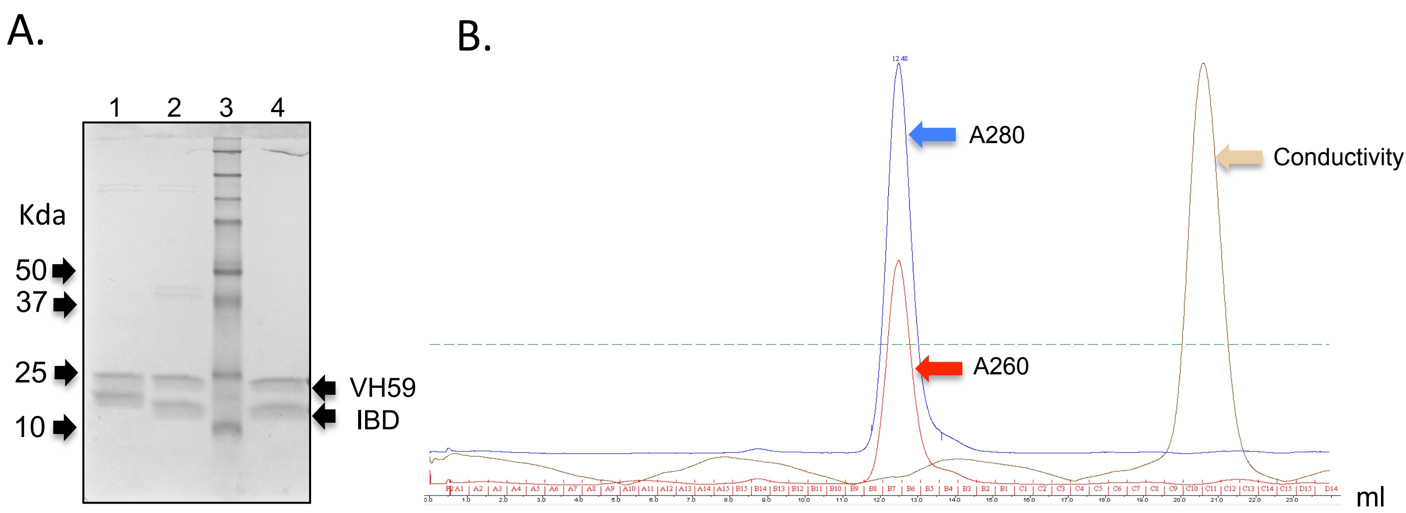

Figure S4. Co-expression and gel filtration of VH59 and LEDGF IBD complex

(A) IBD and VH59 were co-expressed from the T7 promoter induced by IPTG in the pRK-172 vector and the proteins co-purified using nickel agarose beads. The His-IBD and VH59 proteins from the complex are shown in commasie-stained SDS-PAGE in lane 1. TEV protease was used to cleavage the his-tag from the HIS-IBD and after TEV digestion, the proteins were fractionated as shown in lane 2. Nickel agarose beads were used again to remove his-tag to yield the final protein mixture was shown in lane 4. Molecular weight size markers are shown in lane 3.

(**B)** A pre-packed HiLoad Superdex 75 10/300GL column was used to purify the VH59 and IBD complex for crystallization yielding the single eluted peak comprising VH59 and IBD detected at OD_{260} (red) and OD_{280} (blue).

Supplementary Figure 5

ScFv-59 with GST-IBD chip

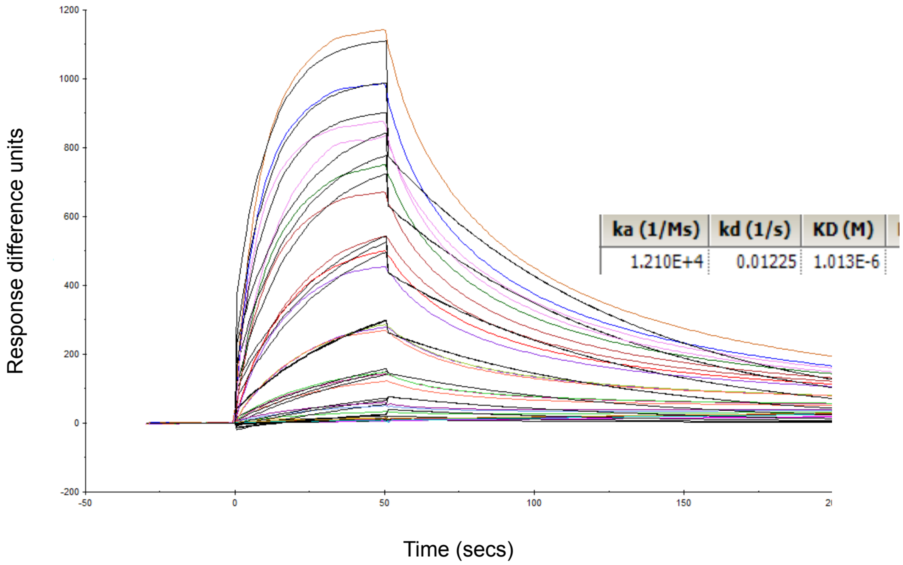

Figure S5. Measurement of anti-IBD scFv-59 affinity using Biacore T200
GST-IBD was bound to the chip using anti-GST antibody with 4000 RU. Sensogrammes of scFv-59 are shown ranging from 6.1 to 0024μM. The Kd was calculated using BIAcore evaluation 2.1 software. The table summarizes values for the association (ka) and dissociation rates (kd), and the calculated equilibrium dissociation constants (KD).

Supplementary Table S1. Crystal data collection and refinement statistics

**Data collection**	
Space Group	P1
Unit cell dimensions	a = 35.0 Å b= 41.4 Å, c = 58.7 Å; α =104.0°, β = 96.1°, γ= 100.5°
Resolution (Å)[a]	19.8-1.7 (1.8-1.7)
Reflections measured/unique	115544/ 30898
R_{meas}[b]	0.083 (0.523)
I/σ	12.2 (2.9)
Completeness (%)	90.4 (92.2)
**Refinement**	
Resolution (Å)	20 - 1.7
Number of molecules per asymmetric unit	2 molecules IBD / 2 molecules VH59
Total number of non-hydrogen protein atoms	3261
Number of water molecules	320
R-factor	0.187 (for 28673 reflections)
R_{free}(%)[c]	0.251 (for 1732 reflections)
R.m.s. deviations:<br>Bond lengths (Å)	 0.018
Bond angles (°)	1.9
Residues in Ramachandran plot (%)[d]<br>Favoured<br>Outliers	 99.0<br>0
Molprobity score[d]	1.30, 97th percentile

a Values in parentheses are for reflections in the highest resolution bin.
b R_{meas} is a redundancy independent R-factor (Diederichs and Karplus, (1997) *Nat. Struct. Biol*. **4**, 269)
c R_{free} was calculated from a subset of 5.1% of the data
d Molprobity (Chen, *et al* (2010) *Acta Cryst D* **66**, 12-21)

Supplementary Table 2:

Contact residues at the IBD-VH59 and IBD-CCD interfaces

**IBD residues**		**VH59 contact residues**		**H-bonds (Å) in VH59-IBD chains H/D and A/E respectively**	**CCD contact residues**
Leu 363	Loop 1-2	Ser 110<br>Phe 111	CDR3<br>CDR3	O-O_{γ} 3.4, 3.3<br>O-N 2.9, 2.9	Gln 168
Lys 364	Loop 1-2	Ser 109	CDR3		Gln 168<br>Ala 169
Ile 365	Loop 1-2	Trp47<br>Pro 108<br>Ser 109<br>Ser 110	CDR3<br>FR<br>CDR3<br>CDR3	 <br>N-O 2.9, 2.9	Gln 168<br>Met 178
Asp 366	Loop1-2				Glu 170<br>His 171<br>Thr 174
Leu 368	Loop1-2	Leu 45	FR		Thr 124
Lys 402	Loop 4-5	Trp 101<br>Leu 112	CDR3<br>CDR3	 N_{ξ}-O 3.3, 3.3	
Arg 405	Loop 4-5	Trp 101<br>Asp 115<br>Trp 117	CDR3<br>FR<br>FR		Trp 131
Phe 406	Loop 4-5	Leu 45<br>Phe 111<br>Trp 117	FR<br>CDR3<br>FR		Trp 131
Val 408	Loop 4-5	Leu 45<br>Tyr 95<br>Trp 117	FR<br>FR<br>FR		Thr 124<br>Lys 127
